# The effect of patient characteristics on second primary cancer risk in France

**DOI:** 10.1186/1471-2407-14-94

**Published:** 2014-02-15

**Authors:** Jérémie Jégu, Marc Colonna, Laetitia Daubisse-Marliac, Brigitte Trétarre, Olivier Ganry, Anne-Valérie Guizard, Simona Bara, Xavier Troussard, Véronique Bouvier, Anne-Sophie Woronoff, Michel Velten

**Affiliations:** 1 Registre des cancers du Bas-Rhin, Laboratoire d’Épidémiologie et de Santé Publique, EA3430, FMTS, Université de Strasbourg, 4 rue Kirschleger, Strasbourg, CEDEX 67085, France; 2 Service de santé publique, Hôpitaux Universitaires de Strasbourg, 1 place de l’hôpital, Strasbourg, CEDEX 67091, France; 3 Registre des cancers de l’Isère, CHU de Grenoble, Pavillon E BP 217, Grenoble, CEDEX 9 38043, France; 4 Registre des cancers du Tarn, 1, rue Lavazière, BP 37, Albi cedex 81001, France; 5 Institut Claudius Regaud, 20–24, rue du Pont-Saint-Pierre, Toulouse cedex 31052, France; 6 Registre des tumeurs de l’Hérault, Centre de Recherche, 208 rue des Apothicaires, Montpellier, CEDEX 5 34298, France; 7 Registre des cancers de la Somme, Service Épidémiologie Hygiène et Santé Publique, CHU Nord, Amiens, CEDEX 1 80054, France; 8 Registre général des tumeurs du Calvados, Cancers & Préventions - U 1086 Inserm, Centre François Baclesse, Avenue du Général Harris BP 5026, Caen, CEDEX 05 14076, France; 9 Registre des cancers de la Manche, Centre Hospitalier Public du Cotentin, 46 rue du Val de Saire, Cherbourg-Octeville 50102, France; 10 Registre des hémopathies malignes de Basse-Normandie, Unité Fonctionnelle Hospitalo-Universitaire n° 0350, Centre Hospitalier Universitaire, Avenue de la Côte de Nacre, Caen, CEDEX 14033, France; 11 Registre des tumeurs digestives du Calvados, Cancers & Préventions - U 1086 Inserm, Centre François Baclesse, Avenue du Général Harris BP 5026, Caen, CEDEX 05 14076, France; 12 Registre des tumeurs du Doubs et du Territoire de Belfort, EA3181, Centre Hospitalier Régional Universitaire Saint-Jacques, Besançon, CEDEX 25030, France; 13 Service d’épidémiologie et de biostatistique, Centre Paul Strauss, 3 rue de la Porte de l’hôpital, Strasbourg, CEDEX 67065, France; 14 Francim : Réseau français des registres des cancers, Toulouse F-31073, France

**Keywords:** Neoplasms, Second primary, Risk assessment, Multivariate analysis, Registries, France

## Abstract

**Background:**

Although cancer survivors are known to be at greater risk of developing second primary cancer (SPC), SPC incidence estimates in France are thus far lacking. We used a multivariate approach to compute these estimates and analyzed the effect of patient characteristics (gender, age at diagnosis, first cancer site, year of diagnosis and follow-up) on SPC risk.

**Methods:**

Data from ten French population-based cancer registries were used to establish a cohort of all patients diagnosed with a first cancer between 1989 and 2004 and followed up until December 31, 2007. The person-year approach was used to estimate standardized incidence ratios (SIRs) and excess absolute risks (EARs) of metachronous SPC. Multivariate Poisson regression models were then used to model SIRs and EARs separately by gender, adjusting for age, year of diagnosis, follow-up and first cancer site.

**Results:**

Among the 289,967 followed-up patients with a first primary cancer, 21,226 developed a SPC. The SIR was of 1.36 (95% CI, 1.35-1.38) and the EAR was of 39.4 excess cancers per 10,000 person-years (95% CI, 37.4-41.3). Among male and female patients, multivariate analyses showed that age, year of diagnosis, follow-up and first cancer site were often independently associated with SIRs and EARs. Moreover, the EAR of SPC remained elevated during patient follow-up.

**Conclusions:**

French cancer survivors face a dramatically increased risk of SPC which is probably related to the high rate of tobacco and alcohol consumption in France. Multivariate modeling of SPC risk will facilitate the construction of a tailored prediction tool to optimize SPC prevention and early detection strategies.

## Background

Cancer survivors are at greater risk of developing second primary cancer (SPC) as well as other diseases [1]. With improvements of cancer survival due to more frequent early detection and advances in cancer treatments, the cancer survivor population continues to grow, reaching an estimated 28.8 million 5-year survivors worldwide [2], surpassing one million in France [2], while the complete US cancer prevalence was estimated to be 11.9 million in 2008 [3]. Although large population-based studies using cancer registries data have been carried out in the US, Italy, Sweden, Switzerland, Australia, England and Wales, Denmark, Finland and Japan to assess the risk of SPC detailed by site of first primary cancer [4-14], SPC incidence estimates in France are thus far lacking.

Moreover, as recent research has highlighted the need to better identify high-risk groups in order to target preventive and interventional clinical strategies, improving knowledge about SPC risks among cancer survivors has become an even more topical issue [15]. In previous population-based studies, even if patient characteristics such as gender, age at diagnosis, first cancer site, year of diagnosis and follow-up have been frequently pointed out to be associated with the risk of SPC, the direct effect of each one of these factors on the risk of SPC has not been assessed simultaneously. This is a matter of concern, as clinicians may be misled while assessing SPC risk of their patients based upon only one of the above-mentioned characteristics, for the simple reason that these characteristics are rarely independent of each other and that confounding is likely to occur. To address this issue, a multivariate analysis of the effects of patient characteristics on SPC risk is indispensable.

The objectives of this study were to compute first SPC incidence estimates in France and to analyze the association between patient characteristics and the risk of SPC, using a multivariate approach.

## Methods

### Data source and management

Data from ten French population-based cancer registries participating in the K2-France nationwide study were used to establish a population-based cohort of all patients presenting with a first cancer diagnosed between 1989 and 2004, excluding non-melanoma skin cancers. Included registries cover eight administrative regions of France (Bas-Rhin, Calvados, Doubs, Hérault, Isère, Manche, Somme and Tarn), which comprise six million inhabitants, representing 9.6% of the metropolitan French population. These registries have achieved a high degree of completeness of ascertainment and incidence data are regularly included in the ‘Cancer Incidence in Five Continents’ monograph series [16]. Since data from these public registries are not publicly available, each registry granted individual permission to use its data for the purposes of this study. The vital status of all patients was updated to December 31, 2007. The proportion of patients lost to follow-up (i.e. alive at some date before December 31, 2007 and with no SPC) was 3.36%.

A SPC was defined as the *first* subsequent primary cancer occurring at least two months (≥61 days) after a first cancer. Extensions, recurrences or metastases were not considered as a SPC according to the IACR rules for multiple primary cancers [17]. Computation of the person-years at risk (PYR) for each individual began after two months of follow-up, and ended at the date of SPC diagnosis, last known vital status, death or December 31, 2007, whichever came first. Patients who developed a synchronous second cancer (<61 days of follow-up) were excluded from this analysis. Data from all available cancer registries were used to determine tumor rank, including data about cancer diagnosed before the 1989–2007 period. Third and subsequent primary cancers were not considered as SPC. Finally, tobacco or alcohol consumption and first cancer treatments were not analyzed because detailed data about these exposures were not available.

The 3rd edition of the International Classification of Diseases for Oncology (ICD-O-3) was used to code invasive tumors [18]. Cancer sites (e.g. head and neck) and subsites (e.g. oral cavity) were defined in accordance with topography and morphology codes used in the EUROCARE project [19]. We focused the analyses on the risk of SPC occurring in a different subsite as the first primary cancer.

### Outcomes

The “standard” person-years approach to study SPC incidence was used [4,20]. The number of observed SPC (O) was compared to the number expected (E) if patients with a first cancer had experienced the same cancer rates as the general population without cancer. Therefore, E was computed by multiplying patient PYRs (allocated by gender, attained age, year of follow-up and first cancer region of diagnosis) with corresponding *first* cancer incidence rates estimated from the general population. Age-Period-Cohort models, where age and cohort effects were modeled using smoothing splines, were utilized to provide robust first cancer incidence rate estimates, especially for scarce sites of cancer [21]. This enables a calculation of the observed to expected ratio (O/E ratio), also called standardized incidence ratio (SIR), which can be interpreted as the relative risk of SPC among patients with a first cancer compared with the general population without cancer. Another relevant indicator provided by this method is the Excess Absolute Rate, commonly called Excess Absolute Risk (EAR), which is the absolute number of excess cancer cases per PYR among cancer patients, obtained by subtracting the expected incidence rate of cancer from the observed incidence rate of SPC.

### Analysis strategy

The first stage of the analysis was to provide univariate estimations of SIR and EAR values of SPC by gender, age, year of diagnosis, follow-up and site of first primary cancer (stratified by gender). Then, patient characteristics associated with the risk of SPC were explored, using multivariate Poisson regression models described by Breslow and Day, Dickman et al. and used recently in the field of SPC incidence [20,22-25].

These models, as opposed to standard Cox regression models, enable account to be taken in the analysis of the number of expected cancers in the general population, and thus a direct modeling of the SIR and the EAR. Ratios of SIRs and ratios of EARs are provided by these models. For example, a ratio of SIR of 1.5 for younger patients compared to older patients means that the SIR is 50% higher among younger patients compared to older ones. In other words, the relative risk of SPC among young patients compared to the general population (SIR) is 50% higher than the relative risk of SPC presented by older patients. SIRs and EARs were modeled separately by gender, adjusting for age, year of first cancer diagnosis, follow-up and first cancer site. Finally, estimates of SIR and EAR values by site of first and second cancer were computed in order to point out which sites deserve special attention during follow-up of cancer survivors.

Byar’s accurate approximation to the exact Poisson distribution was used to compute 95% confidence intervals (95% CI) of SIR and EAR values [20]. With respect to Poisson regression models, assumptions were made that the risk of SPC was constant within each follow-up interval, and that the risk of SPC for any two patient subgroups was proportional over follow-up time. All analyses were performed using SAS version 9.2 (SAS Institute, Cary, North Carolina).

## Results

### Participation flow

A total of 325,407 patients presented with a first cancer between 1989 and 2004 within the area covered by the cancer registries participating in the K2-France study. During the first two months after first cancer diagnosis, 4,053 (1.2%) patients presented a synchronous SPC, 27,190 (8.4%) died without developing a SPC and 4,197 were lost to follow-up (1.3%). Among the remaining cohort of 289,967 patients, 55.5% were males and the mean age at first cancer diagnosis was 64.2 years (SD 15.0). The median follow-up time was 4.0 years.

### Overall SPC risk, univariate estimates

The risk of SPC was increased in this cohort of cancer survivors. With 21,226 patients (7.3%) presenting a SPC occurring in a different subsite as the first primary cancer, compared to 15,555.59 expected on the basis of the rates estimated from the overall population without cancer, the SIR was of 1.36 (95% CI, 1.35-1.38) and the EAR was of 39.4 excess cancers per 10,000 PYR (95% CI, 37.4-41.3).

Univariate estimates of SPC risk differed greatly across patient characteristics, as reported in Table 1. It is worth noting that, despite similar SIR values among male and female patients, males presented more than twice the excess risk of SPC compared with females (EAR of 58.6 versus 21.7), due to a higher expected cancer risk among males in the general population. In a similar way, although higher relative risks of SPC were reported among young patients (SIR of 2.14 for ≤44 years-old patients), cancer survivors facing the highest excess risk of SPC were those who developed a first cancer in the age range 45 to 54 years (EAR = 55.8). Finally, the risk of SPC remained elevated during follow-up, with SIR and EAR values of 1.37 and 42.0 for ≥10 years of follow-up, respectively.

**Table 1:** **Risk of SPC**^**a**^**by gender, age, year of first cancer diagnosis and follow-up, France 1989–2004 (N = 289,967)**

		**O**	**E**	**PYR**	**SIR**	**(95% CI)**	**EAR**^**b**^	**(95% CI)**
**All patients**		21,226	15,555.59	1,440,960	1.36	(1.35-1.38)	39.4	(37.4-41.3)
**Gender**	Male	14,555	10,514.87	689,115	1.38	(1.36-1.41)	58.6	(55.2-62.1)
	Female	6,671	5,040.73	751,845	1.32	(1.29-1.36)	21.7	(19.6-23.8)
**Age at first cancer diagnosis**	≤ 44 y	909	424.88	210,515	2.14	(2.00-2.28)	23.0	(20.2-25.9)
	45 y-54 y	2,592	1,270.67	236,678	2.04	(1.96-2.12)	55.8	(51.7-60.1)
	55 y-64 y	5,284	3,500.56	334,536	1.51	(1.47-1.55)	53.3	(49.1-57.6)
	65 y-74 y	7,772	6,235.67	406,356	1.25	(1.22-1.27)	37.8	(33.6-42.1)
	≥ 75	4,669	4,123.81	252,874	1.13	(1.10-1.17)	21.6	(16.3-26.9)
**Year of first cancer diagnosis**	1989-1994	7,512	5,406.49	517,048	1.39	(1.36-1.42)	40.7	(37.5-44.0)
	1995-1999	7,679	5,634.00	520,856	1.36	(1.33-1.39)	39.3	(36.0-42.6)
	2000-2004	6,035	4,515.10	403,057	1.34	(1.30-1.37)	37.7	(34.0-41.5)
**Follow-up**	≥ 2 m- < 1 y	3,170	2,280.81	213,502	1.39	(1.34-1.44)	41.6	(36.5-46.9)
	≥ 1 y- < 2 y	3,034	2,229.98	210,970	1.36	(1.31-1.41)	38.1	(33.0-43.3)
	≥ 2 y- < 4 y	4,983	3,665.14	343,730	1.36	(1.32-1.40)	38.3	(34.3-42.4)
	≥ 4 y- < 6 y	3,504	2,614.81	241,999	1.34	(1.30-1.39)	36.7	(32.0-41.6)
	≥ 6 y- < 8 y	2,522	1,815.96	166,357	1.39	(1.34-1.44)	42.4	(36.6-48.5)
	≥ 8 y- < 10 y	1,664	1,239.15	112,267	1.34	(1.28-1.41)	37.8	(30.8-45.1)
	≥ 10 y	2,349	1,709.75	152,135	1.37	(1.32-1.43)	42.0	(35.8-48.4)

SPC, second primary cancer; O, Observed; E, Expected; PYR, person-years at risk; SIR, standardized incidence ratio; EAR, excess absolute risk; CI, confidence interval; m, months; y, years.

^a^Excluding SPC occurring in the same subsite as the first primary cancer.

^b^Number of excess cancers per 10,000 person-years at risk.

Results presented in Table 2 show clearly that the risk of SPC was closely tied to the first cancer site, and that patients with a first tobacco and alcohol-related cancer carried the highest increased risk of SPC. Indeed, male patients carried the highest excess risks of SPC if they were diagnosed with a first cancer of the head and neck (EAR = 380.0), larynx (EAR = 266.3), oesophagus (EAR = 232.1), bladder (EAR = 121.7), lip (EAR = 74.3), kidney (EAR = 66.4), lung (EAR = 62.0) or a chronic lymphatic leukaemia (EAR = 50.2). Patients with a first prostate or colorectal cancer presented a rather small excess risk of SPC, with respective EAR values of 16.5 and 15.7. With respect to female patients, the highest excess risks of SPC were noted for first cancer of the larynx (EAR = 205.9), head and neck (EAR = 194.8), oesophagus (EAR = 126.5), vagina and vulva (EAR = 46.2) or an acute myeloid leukaemia (EAR = 42.7). Finally, patients with a first breast or colorectal cancer presented somewhat lower excess risks of SPC (EAR = 16.8 and 16.4, respectively).

**Table 2:** **Risk of SPC**^**a**^**by gender and first cancer site, France 1989–2004 (N = 289,967)**

	**Males**						**Females**					
	**(N = 160, 807)**						**(N = 129,160)**					
**First cancer site**	**O**	**E**	**PYR**	**SIR**	**EAR**^**b**^	**(95% CI)**	**O**	**E**	**PYR**	**SIR**	**EAR**^**b**^	**(95% CI)**
Lip	111	80.66	4,081	1.38*	74.3*	(26.1-129.9)	13	8.44	806	1.54	56.6	(-18.9-171.2)
Tongue and lingual tonsil	329	90.84	6,794	3.62*	350.5*	(299.6-405.8)	41	15.99	1,886	2.56*	132.6*	(71.2-210.2)
Oral cavity	536	129.77	10,034	4.13*	404.9*	(360.6-452.0)	83	23.36	2,863	3.55*	208.3*	(149.3-277.8)
Salivary glands	29	20.45	1,480	1.42	57.8	(-7.0-143.3)	14	12.38	1,569	1.13	10.3	(-30.1-70.8)
Oropharynx	550	129.86	10,157	4.24*	413.7*	(369.3-460.9)	52	14.54	1,813	3.58*	206.6*	(134.0-295.9)
Nasopharynx	13	12.90	1,223	1.01	0.8	(-49.0-76.2)	8	3.33	535	2.41*	87.4*	(2.2-232.6)
Hypopharynx	496	129.23	9,334	3.84*	392.9*	(347.2-441.8)	27	4.40	577	6.13*	391.8*	(232.1-604.9)
Head and neck^c^	1,981	509.07	38,731	3.89*	380.0*	(357.8-403.1)	214	62.40	7,781	3.43*	194.8*	(159.2-234.3)
Oesophagus	290	119.03	7,367	2.44*	232.1*	(188.1-280.1)	27	11.27	1,244	2.40*	126.5*	(52.4-225.2)
Stomach	240	233.16	12,773	1.03	5.4	(-17.7-30.7)	87	83.55	8,739	1.04	3.9	(-15.9-27.2)
Small intestine	32	31.37	1,848	1.02	3.4	(-51.4-74.7)	15	15.39	1,716	0.97	-2.3	(-40.8-54.5)
Colorectum	1,996	1,843.60	97,268	1.08*	15.7*	(6.8-24.9)	935	792.53	87,123	1.18*	16.4*	(9.6-23.5)
Colon	1,185	1,060.19	55,504	1.12*	22.5*	(10.5-35.0)	590	491.42	54,649	1.20*	18.0*	(9.5-27.1)
Rectum	810	783.28	41,747	1.03	6.4	(-6.7-20.2)	344	300.88	32,443	1.14*	13.3*	(2.4-25.1)
Liver, primary	135	120.50	6,749	1.12	21.5	(-10.8-58.2)	23	13.82	1,709	1.66*	53.7*	(4.4-121.1)
Gallbladder and biliary tract	30	38.16	1,950	0.79	-41.9	(-91.9-23.9)	31	24.89	2,463	1.25	24.8	(-15.5-77.6)
Pancreas	33	49.39	2,948	0.67*	-55.6*	(-90.5--10.3)	25	23.53	2,730	1.06	5.4	(-27.0-49.0)
Nasal cavities and sinuses	50	42.29	2,430	1.18	31.7	(-21.3-97.2)	3	5.90	650	0.51	-44.5	(-81.4-44.1)
Larynx	853	323.02	19,900	2.64*	266.3*	(238.0-296.1)	58	16.93	1,995	3.43*	205.9*	(135.9-291.0)
Lung, bronchus and trachea	855	599.96	41,161	1.43*	62.0*	(48.3-76.4)	96	72.95	9,446	1.32*	24.4*	(5.1-46.9)
Pleura	8	13.92	776	0.57	-76.2	(-134.9-23.8)	3	2.45	284	1.22	19.3	(-65.1-222.3)
Bone and cartilages	16	11.53	2,306	1.39	19.4	(-10.4-62.7)	11	8.25	2,087	1.33	13.2	(-13.2-54.8)
Soft tissue	67	55.74	4,965	1.20	22.7	(-7.7-59.1)	39	24.00	3,951	1.62*	38.0*	(9.4-74.2)
Melanoma of skin	303	258.43	20,881	1.17*	21.3*	(5.5-38.6)	288	208.83	32,126	1.38*	24.6*	(14.6-35.6)
Breast	52	45.45	2,282	1.14	28.7	(-29.0-99.6)	2,476	1,885.48	351,434	1.31*	16.8*	(14.1-19.6)
Cervix uteri	-	-	-	-	-	-	265	173.92	28,122	1.52*	32.4*	(21.4-44.4)
Corpus uteri	-	-	-	-	-	-	511	370.65	41,147	1.38*	34.1*	(23.6-45.4)
Ovary and uterine adnexa	-	-	-	-	-	-	189	168.03	22,829	1.12	9.2	(-2.2-21.9)
Vagina and vulva	-	-	-	-	-	-	59	39.51	4,219	1.49*	46.2*	(12.8-86.7)
Prostate	3,880	3,500.52	230,582	1.11*	16.5*	(11.2-21.8)	-	-	-	-	-	-
Testis	86	59.42	19,448	1.45*	13.7*	(4.8-24.1)	-	-	-	-	-	-
Penis	43	42.20	2,303	1.02	3.5	(-48.1-68.3)	-	-	-	-	-	-
Bladder	1,192	729.56	38,002	1.63*	121.7*	(104.1-140.0)	99	79.73	7,772	1.24*	24.8*	(0.9-52.5)
Kidney	660	471.41	28,385	1.40*	66.4*	(49.0-84.9)	198	149.07	17,327	1.33*	28.2*	(12.9-45.3)
Melanoma of choroid	23	19.49	1,361	1.18	25.8	(-36.1-110.4)	14	12.84	1,465	1.09	7.9	(-35.4-72.7)
Brain	24	30.03	5,546	0.80	-10.9	(-26.4-10.2)	20	16.84	4,506	1.19	7.0	(-10.3-31.2)
Thyroid gland	88	62.22	7,369	1.41*	35.0*	(11.3-62.7)	244	174.12	31,397	1.40*	22.3*	(12.8-32.6)
Hodgkin’s disease	69	40.59	8,338	1.70*	34.1*	(15.7-56.0)	38	22.16	6,930	1.72*	22.9*	(6.8-43.3)
Non-Hodgkin’s lymphoma	448	348.23	26,008	1.29*	38.4*	(22.8-55.1)	229	185.26	22,997	1.24*	19.0*	(6.5-32.8)
Multiple myeloma	133	125.42	6,934	1.06	10.9	(-20.3-46.4)	73	66.51	6,946	1.10	9.3	(-13.4-36.4)
Leukaemia	377	323.41	22,217	1.17*	24.1*	(7.4-42.1)	173	130.86	17,269	1.32*	24.4*	(10.0-40.5)
Acute lymphatic leukaemia	4	3.21	3,064	1.25	2.6	(-7.0-23.0)	7	2.91	2,571	2.41	15.9	(-0.4-44.8)
Chronic lymphatic leukaemia	276	219.48	11,261	1.26*	50.2*	(22.1-80.9)	123	91.97	9,091	1.34*	34.1*	(11.3-60.3)
Acute myeloid leukaemia	21	23.63	2,585	0.89	-10.2	(-41.2-32.8)	23	12.37	2,491	1.86*	42.7*	(8.8-88.9)
Chronic myeloid leukaemia	43	47.36	3,291	0.91	-13.3	(-49.4-32.1)	14	16.78	2,234	0.83	-12.4	(-40.9-30.0)
Other sites^d^	542	412.95	25,949	1.31*	49.7*	(32.5-68.1)	211	186.09	22,084	1.13	11.3	(-1.2-25.1)
**All sites**^**d**^	**14,555**	**10,514.87**	**689,115**	1.38*	58.6*	**(55.2-62.1)**	**6,671**	**5,040.73**	**751,845**	1.32*	21.7*	**(19.6-23.8)**

SPC, second primary cancer; O, Observed; E, Expected; PYR, person-years at risk; SIR, standardized incidence ratio; EAR, excess absolute risk; CI, confidence interval.

^a^Excluding SPC occurring in the same subsite as the first primary cancer.

^b^Number of excess cancers per 10,000 person-years at risk.

^c^Head and neck site includes tongue and lingual tonsil, oral cavity, oropharynx, nasopharynx, hypopharynx and other oral cavity and pharynx.

^d^Excluding non-melanoma skin cancer.

*P < .05.

### Multivariate analyses

Among male and female patients, multivariate analyses showed that age, year of diagnosis, follow-up and first cancer site were often independently associated with SIRs and EARs (Table 3). Compared with the second year of follow-up, the EAR for more than 10 years of follow-up was not significantly changed among males (EAR ratio 0.87 95% IC, 0.71-1.07) but was increased among females (EAR ratio 1.83 95% IC, 1.28-2.62). Interestingly, this trend was hidden (or even slightly inverted among males) when considering SIR values, due to the increase of expected rates of cancer with attained age during patient follow-up. These results also show that patients with a first cancer diagnosed at 55 to 64 years-old presented the highest excess risk of SPC. The risk of SPC varied widely across first cancer sites (p < .001) but detailed results are not shown, as the clinical relevance of SIR and EAR ratios across patients with different types of first cancer may be questionable. It should be noted that, although the adjustment on cancer registry showed some heterogeneity between registries (p < .001), this adjustment did not upset the results presented in Table 3 (results not shown).

**Table 3:** **Multivariate Poisson regression models of SPC risk**^**a**^**by gender, France 1989–2004 (N = 289,967)**

		**Males**				**Females**			
		**(N = 160,807)**				**(N = 129,160)**			
		**Ratio of SIRs**	***P***	**Ratio of EARs**	***P***	**Ratio of SIRs**	***P***	**Ratio of EARs**	***P***
		**(95% CI)**		**(95% CI)**		**(95% CI)**		**(95% CI)**	
**Age at first cancer diagnosis**	≤ 44 y	1.98 (1.78-2.20)	<.001	0.63 (0.54-0.75)	<.001	1.27 (1.15-1.41)	<.001	0.54 (0.40-0.72)	<.001
	45 y-54 y	1.49 (1.41-1.59)		0.93 (0.84-1.04)		1.11 (1.03-1.20)		0.90 (0.72-1.13)	
	**55 y-64 y**	**1.00 (ref)**		**1.00 (ref)**		**1.00 (ref)**		**1.00 (ref)**	
	65 y-74 y	0.86 (0.82-0.89)		0.88 (0.78-1.00)		0.93 (0.87-0.99)		0.89 (0.69-1.14)	
	≥ 75	0.81 (0.77-0.85)		0.67 (0.53-0.84)		0.83 (0.77-0.89)		0.51 (0.32-0.81)	
**Year of first cancer diagnosis**	**1989-1994**	**1.00 (ref)**	.271	**1.00 (ref)**	.037	**1.00 (ref)**	.003	**1.00 (ref)**	<.001
	1995-1999	0.97 (0.93-1.01)		0.88 (0.79-0.97)		1.10 (1.04-1.17)		1.48 (1.19-1.84)	
	2000-2004	0.99 (0.95-1.03)		0.92 (0.82-1.04)		1.10 (1.03-1.18)		1.44 (1.10-1.89)	
**Follow-up**	≥ 2 m- < 1 y	1.00 (0.95-1.06)	<.001	0.82 (0.71-0.95)	.037	1.05 (0.96-1.16)	.284	0.93 (0.62-1.33)	.006
	**≥ 1 y- < 2 y**	**1.00 (ref)**		**1.00 (ref)**		**1.00 (ref)**		**1.00 (ref)**	
	≥ 2 y- < 4 y	0.99 (0.94-1.05)		1.02 (0.90-1.17)		1.02 (0.94-1.11)		1.03 (0.76-1.40)	
	≥ 4 y- < 6 y	0.96 (0.90-1.01)		0.96 (0.83-1.12)		1.00 (0.92-1.10)		1.07 (0.76-1.49)	
	≥ 6 y- < 8 y	0.95 (0.89-1.01)		1.01 (0.85-1.20)		1.07 (0.97-1.18)		1.41 (1.01-1.97)	
	≥ 8 y- < 10 y	0.90 (0.83-0.97)		0.90 (0.73-1.11)		1.00 (0.90-1.12)		1.31 (0.89-1.94)	
	≥ 10 y	0.82 (0.76-0.88)		0.87 (0.71-1.07)		1.10 (1.00-1.22)		1.83 (1.28-2.62)	
**First cancer site**		^b^	<.001	^b^	<.001	^b^	<.001	^b^	<.001

SPC, second primary cancer; SIR, standardized incidence ratio; EAR, excess absolute risk; CI, confidence interval; ref, reference category.

^a^Excluding SPC occurring in the same subsite as the first primary cancer.

^b^Detailed results not shown.

### Estimates by site of first and second cancer

Finally, Tables 4 and 5 detail risk estimates of SPC by site of first and second primary cancer, with significant EAR values superior or equal to 3.0 per 10,000 PYR among males and 2.5 among females.

**Table 4:** Risk of SPC by first and second cancer site among male patients, France 1989–2004 (N = 160,807)

**First cancer site**	**Second cancer site**	**O**	**E**	**PYR**	**SIR**^**a**^	**EAR**^**a,b**^	**(95% CI)**
**Lip**	Lung, bronchus and trachea	27	9.52	4,081	2.84	42.8	(20.3-72.9)
	Oesophagus	7	1.79		3.91	12.8	(2.5-31.0)
**Head and neck**	Lung, bronchus and trachea	663	76.15	38,731	8.71	151.5	(138.7-165.1)
	Head and neck^c^	405	24.54		16.50	98.2	(88.3-108.9)
	Oesophagus	269	13.79		19.51	65.9	(57.8-74.7)
	Larynx	76	10.74		7.07	16.8	(12.7-21.8)
	Colorectum	109	60.09		1.81	12.6	(7.6-18.4)
	Liver, primary	62	17.04		3.64	11.6	(7.9-16.1)
	Bladder	53	24.13		2.20	7.5	(4.0-11.7)
	Kidney	35	17.32		2.02	4.6	(1.8-8.1)
	Pancreas	26	11.03		2.36	3.9	(1.5-7.0)
	Stomach	29	14.27		2.03	3.8	(1.3-7.1)
**Oesophagus**	Head and neck	92	6.29	7,367	14.63	116.3	(92.1-144.6)
	Lung, bronchus and trachea	71	16.70		4.25	73.7	(52.6-98.9)
	Larynx	16	2.25		7.11	18.7	(9.4-32.2)
	Colorectum	25	14.51		1.72	14.2	(2.3-30.4)
	Stomach	10	3.72		2.69	8.5	(1.5-19.9)
	Bladder	12	5.96		2.01	8.2	(0.3-20.4)
**Colorectum**	Colorectum^c^	174	119.63	97,268	1.45	5.6	(3.0-8.5)
	Lung, bronchus and trachea	279	240.30		1.16	4.0	(0.7-7.5)
	Bladder	136	104.92		1.30	3.2	(0.9-5.8)
**Larynx**	Lung, bronchus and trachea	310	44.71	19,900	6.93	133.3	(116.5-151.7)
	Head and neck	138	15.78		8.75	61.4	(50.3-74.0)
	Oesophagus	64	7.59		8.43	28.3	(21.0-37.3)
	Bladder	46	16.25		2.83	15.0	(8.8-22.7)
	Colorectum	62	39.40		1.57	11.4	(4.1-20.1)
	Liver, primary	22	10.72		2.05	5.7	(1.5-11.4)
	Stomach	19	9.27		2.05	4.9	(1.1-10.3)
	Pancreas	14	6.96		2.01	3.5	(0.3-8.3)
**Lung, bronchus and trachea**	Head and neck	127	31.83	41,162	3.99	23.1	(18.0-29.0)
	Bladder	105	34.29		3.06	17.2	(12.5-22.5)
	Oesophagus	55	15.65		3.51	9.6	(6.3-13.6)
	Colorectum	121	84.25		1.44	8.9	(3.9-14.7)
	Larynx	45	12.08		3.73	8.0	(5.0-11.7)
	Kidney	45	21.47		2.10	5.7	(2.8-9.4)
	Leukaemia	30	16.07		1.87	3.4	(1.0-6.5)
**Melanoma of skin**	Leukaemia	19	6.22	20,881	3.06	6.1	(2.5-11.2)
**Prostate**	Colorectum	830	692.73	230,582	1.20	6.0	(3.5-8.5)
	Bladder	406	291.02		1.40	5.0	(3.3-6.8)
**Testis**	Kidney	10	2.38	19,448	4.20	3.9	(1.2-8.2)
**Bladder**	Lung, bronchus and trachea	295	94.45	38,002	3.12	52.8	(44.2-62.2)
	Prostate	398	257.66		1.54	36.9	(26.9-47.7)
	Head and neck	55	27.02		2.04	7.4	(3.8-11.7)
	Larynx	28	10.96		2.55	4.5	(2.0-7.8)
	Stomach	38	23.71		1.60	3.8	(0.8-7.5)
**Kidney**	Prostate	269	162.00	28,385	1.66	37.7	(26.7-49.7)
	Bladder	39	24.55		1.59	5.1	(1.1-10.1)
**Thyroid gland**	Prostate	34	20.08	7,369	1.69	18.9	(4.7-37.2)
	Leukaemia	5	1.50		3.33	4.7	(0.1-13.8)
**Hodgkin's disease**	Lung, bronchus and trachea	23	5.72	8,338	4.02	20.7	(10.6-34.5)
	Non-Hodgkin’s lymphoma	6	1.33		4.50	5.6	(1.0-14.1)
	Oesophagus	4	0.93		4.29	3.7	(0.2-11.2)
**Non-Hodgkin’s lymphoma**	Lung, bronchus and trachea	76	46.84	26,008	1.62	11.2	(5.0-18.6)
	Leukaemia	21	8.50		2.47	4.8	(1.7-9.1)
	Bladder	30	17.70		1.70	4.7	(1.0-9.7)
	Stomach	18	10.21		1.76	3.0	(0.2-7.0)
**Chronic lymphatic leukaemia**	Lung, bronchus and trachea	56	27.69	11,261	2.02	25.1	(13.0-40.0)
	Colorectum	41	27.78		1.48	11.7	(1.5-24.7)
	Hodgkin’s disease	4	0.39		10.26	3.2	(0.6-8.7)

SPC, second primary cancer; O, Observed; E, Expected; PYR, person-years at risk; SIR, standardized incidence ratio; EAR, excess absolute risk; CI, confidence interval.

^a^All P < .05.

^b^Number of excess cancers per 10,000 person-years at risk.

^c^SPC occurring in a different subsite as the first primary cancer.

**Table 5:** Risk of SPC by first and second cancer site among female patients, France 1989–2004 (N = 129,160)

**First cancer site**	**Second cancer site**	**O**	**E**	**PYR**	**SIR**^**a**^	**EAR**^**a,b**^	**(95% CI)**
**Head and neck**	Head and neck^c^	57	0.61	7,781	93.83	72.5	(54.7-94.1)
	Lung, bronchus and trachea	51	2.71		18.81	62.1	(45.3-82.7)
	Oesophagus	20	0.41		49.33	25.2	(15.2-39.2)
	Liver, primary	5	0.69		7.25	5.5	(1.2-14.1)
**Oesophagus**	Head and neck	10	0.14	1,244	71.04	79.3	(37.4-146.7)
**Colorectum**	Breast	297	246.36	87,123	1.21	5.8	(2.0-9.9)
	Colorectum^c^	98	67.48		1.45	3.5	(1.4-6.0)
	Corpus uteri	66	41.93		1.57	2.8	(1.0-4.8)
**Liver, primary**	Lung, bronchus and trachea	5	0.61	1,709	8.13	25.7	(5.8-64.7)
	Non-Hodgkin’s lymphoma	3	0.53		5.62	14.4	(0.4-48.2)
	Soft tissue	2	0.06		32.96	11.3	(1.0-41.9)
**Larynx**	Lung, bronchus and trachea	19	0.74	1,995	25.76	91.5	(53.6-145.0)
	Head and neck	10	0.22		45.47	49.0	(22.9-91.1)
	Oesophagus	3	0.11		26.70	14.5	(2.5-43.4)
	Bladder	3	0.28		10.73	13.6	(1.6-42.5)
**Lung, bronchus and trachea**	Head and neck	10	0.99	9,446	10.09	9.5	(4.0-18.4)
	Bladder	6	1.18		5.11	5.1	(1.1-12.6)
	Oesophagus	3	0.47		6.41	2.7	(0.1-8.8)
**Soft tissue**	Ovary and uterine adnexa	4	0.86	3,951	4.68	8.0	(0.6-23.8)
**Melanoma of skin**	Breast	98	75.05	32,127	1.31	7.1	(1.4-13.8)
	Kidney	15	5.32		2.82	3.0	(1.0-6.0)
**Breast**	Corpus uteri	358	147.83	351,434	2.42	6.0	(5.0-7.1)
**Cervix uteri**	Lung, bronchus and trachea	34	7.66	28,122	4.44	9.4	(5.6-14.2)
	Colorectum	40	22.60		1.77	6.2	(2.1-11.3)
	Vagina and vulva	10	1.28		7.82	3.1	(1.2-6.1)
	Leukaemia	12	4.17		2.88	2.8	(0.7-6.0)
	Bladder	10	2.45		4.08	2.7	(0.8-5.7)
**Corpus uteri**	Breast	174	120.03	41,147	1.45	13.1	(7.1-19.9)
	Colorectum	106	60.33		1.76	11.1	(6.4-16.5)
	Lung, bronchus and trachea	34	17.06		1.99	4.1	(1.6-7.4)
**Vagina and vulva**	Cervix uteri	6	0.68	4,219	8.87	12.6	(3.6-29.4)
	Bladder	4	0.88		4.53	7.4	(0.5-22.2)
	Head and neck	3	0.47		6.37	6.0	(0.3-19.7)
**Kidney**	Breast	62	46.58	17,327	1.33	8.9	(0.6-19.0)
	Lung, bronchus and trachea	16	6.61		2.42	5.4	(1.5-11.2)
	Leukaemia	11	4.07		2.70	4.0	(0.8-9.0)
	Thyroid gland	8	2.70		2.96	3.1	(0.4-7.5)
**Thyroid gland**	Leukaemia	13	3.95	31,397	3.29	2.9	(0.9-5.8)
**Hodgkin’s disease**	Leukaemia	5	0.50	6,930	10.05	6.5	(1.6-16.1)
	Non-Hodgkin’s lymphoma	5	0.73		6.89	6.2	(1.3-15.8)
	Lung, bronchus and trachea	4	0.97		4.14	4.4	(0.2-13.4)
**Non-Hodgkin’s lymphoma**	Stomach	12	5.02	22,997	2.39	3.0	(0.5-6.9)
	Kidney	12	4.99		2.41	3.0	(0.5-6.9)
**Chronic lymphatic leukaemia**	Breast	41	26.30	9,091	1.56	16.2	(3.4-32.3)
**Acute myeloid leukaemia**	Ovary and uterine adnexa	3	0.48	2,491	6.26	10.1	(0.5-33.3)

SPC, second primary cancer; O, Observed; E, Expected; PYR, person-years at risk; SIR, standardized incidence ratio; EAR, excess absolute risk; CI, confidence interval.

^a^All P < .05.

^b^Number of excess cancers per 10,000 person-years at risk.

^c^SPC occurring in a different subsite as the first primary cancer.

There were consistent and reciprocal associations between cancer of the head and neck, oesophagus, larynx and lung. Bi-directional risks between lung and bladder cancers were found. Bladder cancers were more frequent after a first head and neck, oesophagus, larynx and kidney cancer.

There were significant associations between cancers of the upper and lower digestive tract (head and neck, oesophagus and colorectum). Among women, there was a bidirectional association between breast and corpus uteri cancers. Among men, patients with a first thyroid cancer presented an increased risk of prostate cancer. There was an association between colorectal and bladder cancers in men and between colorectal and corpus uteri cancer in women. Patients with a first urological cancer (bladder, kidney) presented a dramatically increased risk of prostate SPC. Finally, patients presented an increased risk of lung cancer after Hodgkin’s lymphoma among males, kidney cancer after testicular cancer, colorectal or bladder cancer after prostate cancer or cervical cancer.

## Discussion

### Main findings of this study

Firstly, French cancer survivors face a 36% increased risk of SPC relative to the overall population without cancer (SIR = 1.36). This risk is markedly high compared with published estimates for Denmark (SIR = 0.91), Italy (0.93), Finland (1.12), US (1.14), Vaud Canton, Switzerland (1.2), Osaka Prefecture, Japan (1.21) and Queensland, Australia (1.27) [4-10]. A strong argument that can be put forward to explain this singular position of France is the particularly high alcohol and tobacco consumption among male adults in France. Indeed, although alcohol consumption decreased in France from 25.0 to 13.7 l of pure alcohol per capita between 1961 and 2004, this consumption remains still high compared with the US consumption level of about 9.4 l per capita over the same period [26]. In addition, estimated age-standardized prevalence of current smoking among male adults was of 36% in 2006 in France, compared with 25% in the US [27]. Finally, in this cohort of French survivors, tobacco and/or alcohol-related cancer sites accounted for more than 50% of the total excess SPC, compared with 35% in the US, as estimated by the Surveillance, Epidemiology, and End Results (SEER) Program [4].

Secondly, multivariate analyses showed that most patient characteristics were independently associated with SIRs and EARs, and that the EAR of SPC remained elevated during patient follow-up. Although previous studies stressed the slightly decreasing trend of SIRs with follow-up [4], the elevated level of the EARs of SPC during follow-up has never been clearly identified, due primarily to the concurrent increase of the expected rates of cancer with attained age. The effect of follow-up on SPC risk may be the result of two distinct reverse effects; on the one hand the extension of carcinogen exposure after first cancer diagnosis strengthens the risk of SPC and, on the other hand, the progressive selection through cancer survival of patients with a more favorable morbidity profile lowers the risk of SPC. Pancreatic cancer is an extreme example of this progressive selection of patients through survival, pancreatic cancer survivors being significantly “protected” against SPC compared with the general population without cancer (SIR = 0.67 in our cohort). This may explain the apparent contradictory results observed among men and women in our study as, considering that survival is often worse among men compared to women in France [28], the higher selection of male patients with favorable profile during follow-up may have outweighed the strengthening effect of the extension of carcinogen exposure on the risk of SPC.

### Patterns of first and second primary cancers

To date, different explanations have been put forward in the literature to explain the specific patterns of first and second primary cancers among cancer survivors. These include high tobacco and/or alcohol consumption, hormonal and nutritional factors, exposure to virus and immunosuppression, genetic predisposition, increased site-specific medical surveillance, late adverse effects of first cancer treatments and interactions among these factors [4,5,15].

#### High tobacco and/or alcohol consumption

We confirmed consistent and reciprocal associations between cancer of the head and neck, oesophagus, larynx and lung related to high tobacco and alcohol exposure [4,25,29-31]. Other cancers that may be related to a high tobacco and/or alcohol consumption include those of the bladder, lip, stomach, liver, pancreas, kidney, cervix uteri, colon and rectum. According to the concept of “field cancerization”, factors such as an exposure to carcinogens induce a field of mucosa more susceptible to tumor formation and, due to the common conduit connecting the upper aero-digestive tract, this is believed to elevate epithelial cancer risk through the head and neck, lung and oesophagus [31-33]. These associations, although well-recognized in the literature, were strikingly pronounced in our dataset. For instance, among male patients with a head and neck cancer, respective EAR of lung, head and neck and oesophagus SPC were 151.5, 98.2 and 65.9 per 10,000 PYR, while EAR estimates provided by the SEER Program were 59.8, 75.2 and 14.2. These stronger associations may be a consequence of the higher exposure to alcohol (decrease in alcohol consumption from 25.0 to 13.7 l of pure alcohol per capita between 1961 and 2004 in France compared with 9.4 l over the same period in the US) and tobacco (prevalence of current smoking among males of 36% in France, compared with 25% in the US in 2006) in our cohort [26,27].

Although there is growing evidence about the adverse effects of persistent tobacco smoking on SPC risk, treatment effectiveness and survival, up to one-third to one-half of patients continue to smoke after diagnosis or relapse after initial quit attempts, depending on tumor site and duration of follow-up [34]. In particular, among patients with a head and neck cancer, persistent tobacco smoking and alcohol consumption following index cancer diagnosis has been reported to range from 30 to 40% and 34 to 44%, respectively [35]. Continued tobacco smoking and/or alcohol drinking after treatment have been estimated to be responsible for one-third of the SPCs of patients with an index head and neck cancer [36]. Finally, it appears that cancer diagnosis is underused as a teachable moment for smoking cessation and that more research is needed to empirically test and adapt smoking and alcohol drinking cessation interventions for cancer patients [1,34,37].

#### Hormonal and nutritional factors

Identified hormonal-related cancers in the literature include breast, prostate, endometrium, testis, ovary, thyroid and osteosarcoma [38]. In our cohort, we confirmed among women a bidirectional association between breast and corpus uteri [4,9]. Among men, we only found a unidirectional association between thyroid and prostate cancer [4].

Concerning nutritional factors, higher body-mass index (BMI) is strongly associated with oesophageal adenocarcinoma, thyroid, colon and rectum cancers in men, and endometrial, gallbladder, oesophageal adenocarcinoma and renal cancers in women [39]. To date, the main mechanisms proposed to explain these associations involve hormonal systems interlinked through insulin [39]. Although data about BMI were not available, it is likely that overweight and obesity played a role in the associations between cancers of the upper and lower digestive tract (head and neck, oesophagus and colorectum) observed in our cohort, as reported in a previous study among colorectal cancer patients performed in the French region of Isère [40]. Leaving to one side tobacco and alcohol consumption, low intake of fruits and vegetables may also have contributed to the excess risk of SPC observed in the upper and lower digestive tract, as previously reported [4].

#### Exposure to virus and immunosuppression

It is established that Epstein-Barr virus, human papilloma virus (HPV), hepatitis C virus, hepatitis B virus, human herpes virus 8 and human T-cell leukemia virus are causative agents of human cancer [41] and may have contributed to certain associations of cancers. For example, in our cohort, 12.5% of colorectal SPCs after a first cervix uteri cancer were anal tumors, which could be a consequence of HPV infections and underlying immunologic defects. Oropharyngeal squamous cell cancer of the lingual and palatine tonsils recorded in our cohort may also be related to HPV infection [42]. Moreover, human immunodeficiency virus or alteration of the regulation and inflammation systems may also have contributed to certain excess cancers observed after first lymphoproliferative tumors [4].

#### Genetic predisposition

Cancer survivors with a family history of cancer may be at high risk of SPC, with most common syndromes identified to date including mutations in BRCA1 and BRCA2 genes and Lynch syndrome [15]. Carriers of BRCA1 or BRCA 2 tumor suppressor gene mutations face a high lifetime risk of breast and ovarian cancer, as well as a moderate increase in the risk of pancreatic, uterine body and uterine cervix cancers for BRCA1 [43], and malignant melanoma, pancreatic, stomach, gallbladder and bile duct cancers for BRCA 2 [44]. We retrieved no further evidence of these associations in our population-based cohort, considering that patients with an ovarian cancer did not present a significantly increased risk of SPC, while an association was only found between breast and corpus uteri cancer. This may be explained by the low prevalence of these mutations in the population. Indeed, Malone et al. reported a respective prevalence of 2.4% and 2.3% for mutations of BRCA1 and BRCA2 in a population-based study of breast cancer in white and black American women aged 35 to 64 years [45].

Lynch syndrome, also called hereditary nonpolyposis colorectal cancer, is due to a mutation in one of the DNA-mismatch repair genes and is characterized by the development of cancer of the colorectum, endometrium and, less frequently, cancer of the small bowel, stomach, urinary tract, ovaries and brain [46]. We effectively found an association between colorectal and bladder cancers in men and between colorectal and corpus uteri cancer in women. However, considering the low prevalence of this syndrome (1-5% of colorectal cancers) [46], it is unlikely that such genetic predispositions have outweighed the impact of tobacco and alcohol, hormonal and nutritional factors.

#### Increased site-specific medical surveillance

We confirmed in our cohort that patients with a first urological cancer (bladder, kidney) present an increased risk of prostate SPC [5]. Although some prostate SPC may have been discovered consecutive to a radical cystoprostatectomy for first bladder cancer treatment, it cannot be excluded that a non-negligible part of these SPC may have been diagnosed through repeated PSA testing during follow-up.

#### Late adverse effects of first cancer treatments

Curtis et al. reported that, by contrast to children and young adults, cancer therapy among older adults was not associated with a substantial increase in subsequent cancer risk [4]. Indeed, radiotherapy-associated cancers include a long latency period of at least 5 to 10 years and a tendency to arise within or at the edges of prior treatment fields [15]. Association confirmed in our study include lung cancer after Hodgkin’s lymphoma [4,47,48], kidney cancer after testicular cancer [49], colorectal or bladder cancer after prostate cancer [50] or cervical cancer [51]. However, it should be noted that, among US adults, the estimated proportion of SPC attributable to radiotherapy was 8%, which finally suggests that most of them are due to other factors, such as lifestyle or genetics [52].

With respect to chemotherapy-related malignancies, the use of alkylating agents or drugs binding to the enzyme DNA-topoisomerase II may have contributed to subsequent leukemia after Hodgkin’s disease in women and non-Hodgkin’s lymphoma in men reported in our study [53]. Among breast cancer patients, hormonal therapy (primarily tamoxifen) may have contributed to the increase in the subsequent risk of uterine cancer observed in our cohort [54].

#### Interactions among these factors

To date, few studies have enlightened the interactions between risk factors of SPC. The International Head and Neck Cancer Epidemiology consortium reported that simultaneous exposure to tobacco and alcohol involved a greater than multiplicative joint effect on the risk of head and neck cancer [55]. Finally, smoking increases the risk of radiotherapy-related or alkylating agent-related lung SPC in patients treated for a Hodgkin’s disease [48,56].

### Methodological considerations

Several methodological aspects of this analysis require special attention. A first questionable point is the 2-month delay used to define metachronous tumors. Although this limit was consistent with the one used in most previous population-based studies of this type [4-7,10], we performed sensitivity analyses using a 6-month delay, which provided very similar results (SIR = 1.35 and EAR = 38.3 versus SIR = 1.36 and EAR = 39.4, as reported in our study).

Secondly, we excluded patients with a synchronous tumor. This is slightly different from the exclusion process of synchronous cancers performed in other studies, where patients with two synchronous cancers could be included, with any subsequent cancer (i.e. third, forth, …) being considered as a new primary [4,5]. The impact of the inclusion of patients with synchronous SPC may lead, by an anticipation of prevalence cases during the synchronous period, to:

– on the one hand, a decrease of the number of cases that may have been observed in subsequent follow-up periods or,

– on the other hand, to keeping patients who present a particularly high carcinogenic exposition and thus carry the highest risk of new malignancy.

We repeated our analysis by including the 4,053 patients (1.2%) with synchronous SPC and considered any third cancer developed by these patients as a SPC. We found respective SIR and EAR values of 1.37 and 40.0, which shows that including patients with synchronous cancer may have resulted in an only slight overestimation of the risk of SPC.

A third questionable point may be the consideration of the activity period of cancer registries and its impact on cancer rank determination. Indeed, more recently established registries may wrongly consider some SPC as first cancers if the true first cancer diagnosis date is anterior to the beginning of the registration period. In order to appreciate the direction and the magnitude of this bias, we conducted sensitivity analyses in Bas-Rhin, comparing overall SPC risk estimates obtained over fictional registration periods beginning ten years (1979), five years (1984) and zero years (1989) before the study period 1989–2007. We found that, with respect to the ten-year period, SIRs were overestimated by 0.1% and 0.6% for the five-year and zero-year periods, respectively. In other words, considering some SPC as first cancers leads to a small overestimation of SPC risk by keeping patients with higher risk of new malignancies evidenced by the presence of two cancers. In our study, almost all cancer registries were established from 1975 to 1983 (i.e. more than five years before 1989), with the exception of Manche cancer registry, set up in 1994. Considering that cases from the Manche cancer registry only represent 7.4% of our cohort, the overall magnitude of this bias is very small.

Fourthly, new malignancies occurring among patients who migrate from their original cancer registry geographic area may not have been recorded. According to census data, the mean annual migration rate out of any French administrative region (called “département”) was estimated as 3.1% between 1999 and 2004, decreasing with age from 6.9% to 1.2% for 20–29 and ≥ 60 years-old people, respectively [57]. Considering that the mean age at first cancer diagnosis is superior to 60 years in our cohort, it is reasonable to assume that this migration bias, although more significant among young patients, would not upset the overall results presented here. Moreover, a recent study assessing this type of bias among US childhood cancer survivors found no evidence of an outmigration bias in the incidence of SPCs in SEER [58].

Finally, we focused the analyses on the risk of SPC occurring in a different subsite as the first primary cancer, and this for two reasons. First, SPCs arising in the same subsite of non-paired organs are scarce, mainly due to surgical removal of the affected organ [4]. This is a well-known issue while considering prostate cancer survivors, as the high number of expected (E) SPC of the prostate calculated in such an elderly population is largely overestimated compared with the real number of prostate SPC likely to occur among these patients. Second, according to the common set of rules proposed by the International Agency for Research on Cancer (IARC), multiple primaries arising in the same site are only to be recorded when the morphology belongs to different groups [17]. For instance, among breast cancer patients, the number of observed (O) subsequent breast cancers may be underestimated as most of the new malignancies of the breast will not be recorded because of similar morphology. Ultimately, these respective overestimations of E and underestimations of O would both have led to underestimated SIR and EAR values, which rendered it advisable to exclude cancers occurring in the same subsite from SPC risks calculations. It should be noted that such exclusion was also performed in recent studies about SPC incidence performed in the US (an “all excluding same site” estimate of the risk of subsequent cancers is provided) and in the Japanese region of Osaka [4,9].

### Future directions

Considering the dramatically high risk of tobacco and alcohol-related SPC in France, the results of this study will be of immediate use in a public health perspective for the preparation of the third French Cancer Plan. In particular, strengthening the measures to control tobacco and alcohol epidemics in France and promoting research about smoking cessation and alcohol abstinence interventions adapted to cancer survivors should be considered.

The use of multivariate models to analyze SPC incidence with cancer registry data will provide new research perspectives. Indeed, for specific sites of first cancer which present a particularly high risk of SPC identified in this overall analysis, further analyses are in progress in the framework of the K2-France study to provide detailed results about follow-up strategy and etiological research questions.

Furthermore, as there are no individual-specific risk prediction tools akin to those used for selected primary cancers [15], the use of the multivariate approach described in this paper will allow a determination of which clinical characteristics are associated with the risk of SPC for a given site of first cancer, enabling the construction of a tailored risk prediction tool. Such a tool will facilitate SPC individualized risk assessment by clinicians and contribute to the implementation of optimal prevention and early detection strategies.

## Conclusions

French cancer survivors face a dramatically increased risk of SPC which is probably related to the high rate of tobacco and alcohol consumption in France. Multivariate modeling of SPC risk will facilitate the construction of a tailored prediction tool to optimize SPC prevention and early detection strategies.

## Abbreviations

CI: Confidence interval; E: Expected; EAR: Excess absolute risk; IARC: International Agency for Research on Cancer; O: Observed; PYR: Person-years at risk; SEER Program: Surveillance, Epidemiology, and End Results Program; SIR: Standardized incidence ratio; SPC: Second primary cancer.

## Competing interests

The authors declare that they have no competing interest.

## Authors’ contribution

JJ and MV conceived the study, participated in its design and coordination and drafted the manuscript. JJ performed the statistical analysis. MC, LDB, BT, OG, AVG, SB, XT, VB, ASW and MV contributed to the acquisition of data and critically revised the manuscript for important intellectual content. All authors read and approved the final manuscript

## Pre-publication history

The pre-publication history for this paper can be accessed here:

http://www.biomedcentral.com/1471-2407/14/94/prepub
